# Safety profile and risk factors for bleeding in transbronchial cryobiopsy using a two-scope technique for peripheral pulmonary lesions

**DOI:** 10.1186/s12890-021-01817-8

**Published:** 2022-01-10

**Authors:** Toshiyuki Nakai, Tetsuya Watanabe, Yuto Kaimi, Koichi Ogawa, Yoshiya Matsumoto, Kenji Sawa, Atsuko Okamoto, Kanako Sato, Kazuhisa Asai, Yuji Matsumoto, Masahiko Ohsawa, Tomoya Kawaguchi

**Affiliations:** 1 Department of Respiratory Medicine, Graduate School of Medicine, Osaka City University, 1-4-3 Asahi-machi, Abeno-ku, Osaka, 545-8585 Japan; 2 Department of Pathology, Graduate School of Medicine, Osaka City University, 1-4-3 Asahi-machi, Abeno-ku, Osaka, 545-8585 Japan; 3 Department of Endoscopy, Respiratory Endoscopy Division, National Cancer Center Hospital, 5-1-1, Tsukiji, Chuo-ku, Tokyo, 104-0045 Japan; 4 Department of Thoracic Oncology, National Cancer Center Hospital, 5-1-1, Tsukiji, Chuo-ku, Tokyo, 104-0045 Japan

**Keywords:** Bronchoscopy, Peripheral pulmonary lesion, Lung cancer, Cryobiopsy, Two-scope technique

## Abstract

**Background:**

A balloon occlusion technique is suggested for use in cryobiopsy for interstitial lung diseases because of the bleeding risk. However, it may interfere with selection of the involved bronchus for peripheral pulmonary lesions (PPLs). A two-scope technique, in which two scopes are prepared and hemostasis is started using the second scope immediately after cryobiopsy, has also been reported. This study aimed to evaluate the safety and diagnostic utility of transbronchial cryobiopsy using the two-scope technique for PPLs.

**Methods:**

Data of patients who underwent conventional biopsy followed by cryobiopsy using the two-scope technique for PPLs from November 2019 to March 2021 were collected. The incidence of complications and risk factors for clinically significant bleeding (moderate to life-threatening) were investigated. Diagnostic yields were also compared among conventional biopsy, cryobiopsy, and the combination of them.

**Results:**

A total of 139 patients were analyzed. Moderate bleeding occurred in 25 (18.0%) patients without severe/life-threatening bleeding. Although five cases required transbronchial instillation of thrombin, all bleeding was completely controlled using the two-scope technique. Other complications included two pneumothoraces and one asthmatic attack. On multivariable analysis, only ground-glass features (*P* < 0.001, odds ratio: 9.30) were associated with clinically significant bleeding. The diagnostic yields of conventional biopsy and cryobiopsy were 76.3% and 81.3%, respectively (*P* = 0.28). The total diagnostic yield was 89.9%, significantly higher than conventional biopsy alone (*P* < 0.001).

**Conclusions:**

The two-scope technique provides useful hemostasis for safe cryobiopsy for PPLs, with a careful decision needed for ground-glass lesions.

## Background

Lung cancer is the leading cause of cancer-related deaths worldwide. Since the widespread use of low-dose computed tomography (CT) screening has promised to reduce lung cancer mortality, the detection and diagnosis rates of peripheral pulmonary lesions (PPLs) have increased [1, 2]. PPLs have a high incidence of early-stage lung cancer and can also represent a wide variety of benign conditions [3]. Therefore, precise pathological confirmation for PPLs with high-quality tissue specimens obtained using non-surgical biopsy is preferred to avoid unnecessary surgical resections [4, 5]. In addition, even in the case of advanced-stage disease, accounting for 65% of non-small cell lung cancer (NSCLC) [6], adequate amounts of tissue specimens without crush artifacts are required, because identification of the tumor’s immunohistochemical and molecular characteristics is necessary for its appropriate management [7, 8]. However, obtaining large and well-preserved specimens using conventional biopsy during bronchoscopy, a minimally invasive and well-established procedure for diagnosing PPLs, can be difficult and leads to severe clinical limitations for determining treatment plans for both early and advanced stages [9, 10].

Cryobiopsy is emerging as a relatively new technique for collecting larger tissue specimens with fewer crush artifacts during bronchoscopy compared to conventional biopsy [11, 12]. Its diagnostic utility for interstitial lung diseases (ILDs) and endobronchial malignancies has been reported [13, 14]. Moreover, the utility of the quantitative and pathological advantages of cryobiopsy for treatment plans in lung cancer may facilitate the use of cryobiopsy for PPLs [15]. However, the technique has been acknowledged to be challenging for PPLs, because the optimal technique to control bleeding caused by cryobiopsy is unknown. Bleeding is one of the main complications associated with cryobiopsy [16]. Although the prophylactic balloon occlusion technique has generally been used as a hemostatic technique for cryobiopsy of ILDs [17], preventive placement of the balloon during the procedure is detrimental in PPLs because it interferes with bronchoscope manipulation and guiding of the devices such as radial endobronchial ultrasound (R-EBUS), guide sheath (GS), and the cryoprobe into the correct bronchus route toward the targeted lesion. Thus, other ideal and feasible techniques for both bleeding control and endoscopic maneuverability are required to use cryobiopsy for PPLs. In addition, to reduce severe hemorrhage associated with cryobiopsy for PPLs, it is essential to confirm the risk factors for bleeding.

The two-scope technique is an alternative method of hemostasis for cryobiopsy of ILDs; it has established safety and diagnostic utility [18]. It allows physicians to control bleeding promptly and manipulate devices flexibly during the procedure. This study evaluated the safety and diagnostic utility of transbronchial cryobiopsy using the two-scope technique for PPLs. To further assess safety with respect to bleeding, predictive factors for clinically significant bleeding due to cryobiopsy were also examined.

## Methods

### Patients

The medical records of consecutive patients who underwent cryobiopsy using the two-scope technique for PPLs suspected to be lung cancer at Osaka City University Hospital between November 2019 and March 2021 were retrospectively reviewed. The study was approved by the Ethics Committee of Osaka City University Hospital (protocol no. 2021-056). Written informed consent was obtained from all patients prior to bronchoscopy.

### Bronchoscopy equipment and conventional biopsy procedure

All bronchoscopies were performed using a bronchoscope (BF-P260F/BF-P290 as a thin scope or BF-1T260/BF-1TQ290 as a thick scope, Olympus, Tokyo, Japan) in combination with an R-EBUS probe (UM-S20-17S or UM-S20-20R, Olympus) and a cryoprobe (1.9 mm 20402-040 or 2.4 mm 20402-032, Erbe Elektromedizin GmbH, Germany) under local anesthesia with a combination of pethidine hydrochloride and midazolam for conscious sedation. A 2.55-mm GS kit (K-203, Olympus) was used when the thick scope was selected. A 1.9-mm cryoprobe was used through the working channel of the thin scope or the GS combined with the thick scope. A 2.4-mm cryoprobe was used with the thick scope after removing the GS. Virtual bronchoscopy (VB) and virtual fluoroscopy (Ziostation2®, Ziosoft Ltd., Tokyo, Japan) were created using thin-section CT (TSCT) images [19, 20]. The type of bronchoscope and the size of cryoprobe were selected by each operator based on CT images and VB navigation. Though treatment with antiplatelet or anticoagulant agents was discontinued before bronchoscopy, bridging anticoagulation therapy was performed in patients with high thromboembolic risk [21].

Before the procedures, all patients were intubated fiberoptically with an 8.0- or 8.5-mm uncuffed endotracheal tube (Portex® Siliconised PVC Oral/Nasal Uncuffed Tracheal Tube, Smiths Medical, Minneapolis, MN, USA). An R-EBUS probe was inserted through the working channel of the bronchoscope and advanced towards the target PPL by referring to VB, virtual fluoroscopy, and X-ray fluoroscopy (VersiFlex VISTA®, Hitachi, Japan). When assessing the location of the R-EBUS probe against the target lesion, the images were categorized as “within”, “adjacent to”, or “invisible” [22]. After detecting the target lesion, conventional biopsies using a forceps (FB-15C-1 or FB-231D, Olympus) or an aspiration needle (NA-1C-1, Olympus) were performed. Forceps biopsies were repeated 5–10 times to collect tissue samples [23, 24]. The occurrence of pneumothorax was routinely checked by fluoroscopy at every biopsies.

### The two-scope technique for cryobiopsy

**Fig. 1:**
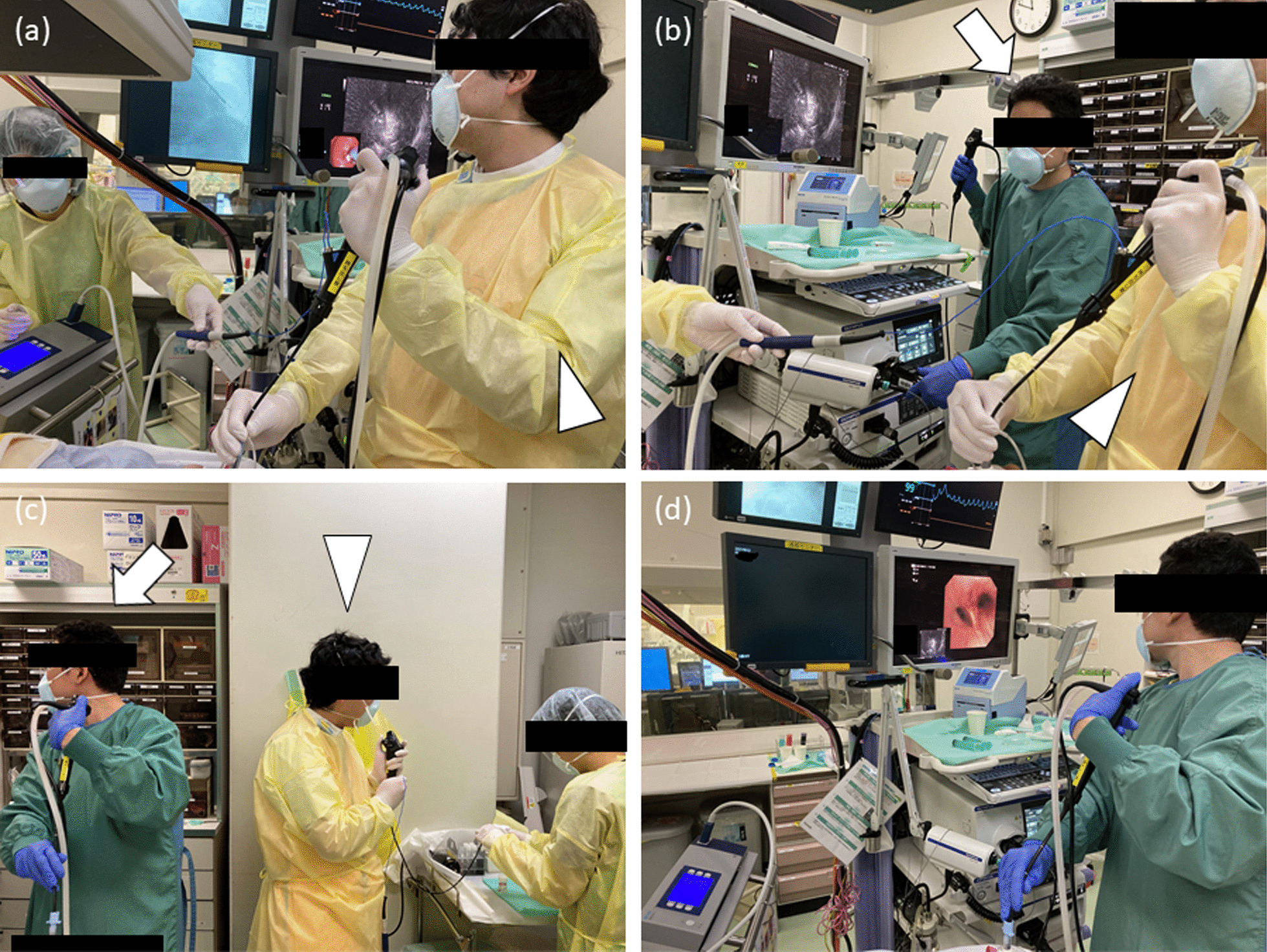
Transbronchial cryobiopsy using the two-scope technique for a peripheral pulmonary lesion. After passing the cryoprobe to the target lesion, the bronchoscope’s tip is wedged and fixed into the involved bronchus under X-ray fluoroscopy by the operator (arrowhead) (**a**). The bronchoscope for cryobiopsy is detached from the light source while the operator (arrowhead) keeps the cryoprobe with the bronchoscope in place, and an assistant (arrow) connects the second thick scope for hemostasis to the light source and suction tube (**b**). Cryobiopsy is performed under fluoroscopic guidance by the operator (arrowhead), and an assistant (arrow) immediately inserts the second thick scope through the endotracheal tube (**c**). An assistant controls bleeding, and the patient is simultaneously rotated to the biopsy-side down lateral decubitus position (**d**)

After completing conventional biopsies, cryobiopsy using the two-scope technique was subsequently performed at the same sites as the conventional biopsies. The technique was modified a little to a previously reported method to suit cryobiopsy for PPLs [18] (Fig. 1). After guiding the cryoprobe through the bronchoscope towards the target lesion, the tip of the scope was wedged as close as possible to the target lesion under fluoroscopic guidance. The connector of the scope was then detached from the light source of an endoscope system while the operator kept the cryoprobe in place. Next, an assistant connected the second thick scope to control bleeding to the light source. The suction tube was also detached from the first scope for cryobiopsy and connected to the second scope for hemostasis. The final adjustment of the cryobiopsy site was made by matching the views of anterior–posterior and 45-degree right/left anterior oblique between X-ray fluoroscopy and virtual fluoroscopy. After the assistant was ready for hemostasis, the cryoprobe was cooled for 3–6 s. The tissue sample attached to the frozen tip was removed by extracting the cryoprobe together with the first scope by the operator. The second scope was then immediately inserted towards the cryobiopsy site by the assistant, and the patient was simultaneously rotated to the biopsy-side down lateral decubitus position. Hemostatic agents were injected when bronchoscopic suction alone was insufficient to stop bleeding. The collected tissue samples were thawed in normal saline and fixed in formalin. Rapid on-site cytologic evaluation was optional. The number of conventional biopsies and cryobiopsies was determined during the procedure in the discretion by the operator and the assistant, based on the amount of bleeding after each biopsy, the size of the obtained tissue sample, the results of the rapid on-site cytologic evaluation, and whether next-generation sequencing analysis was performed. Chest radiography was routinely performed at least until the next day to assess complications.

### Complications

The following complications associated with the procedure were reviewed: bleeding, pneumothorax, pulmonary infection, hypoxia, respiratory failure, and other life-threatening events. The severity of bleeding was determined on a scale of four steps according to the standardized definitions of bleeding after transbronchial lung biopsy [25]: mild, bleeding requiring less than 1 min of suctioning or wedging of the bronchoscope; moderate, bleeding requiring more than 1 min of suctioning or wedging or instillation of cold saline, diluted vasoactive substances, or thrombin; severe, bleeding requiring selective intubation using an endotracheal tube or balloon/bronchial blocker for less than 20 min; and life-threatening, bleeding requiring persistent selective intubation for more than 20 min, blood transfusion, bronchial artery embolization, or intensive care therapy. Moreover, moderate to life-threatening was defined as clinically significant bleeding.

### Diagnostic criteria

The final diagnosis was determined based on the histological findings. Histological findings of malignancy were considered diagnostic. Cases of benign conditions were considered diagnostic when specific benign findings (i.e., granuloma, fibrosis, and inflammation) on histology or positive microbiological cultures were found and the subsequent clinical courses were compatible after a 6-month follow-up period. Cases without significant histological and/or microbiological findings and that decreased in size after at least 6 months of follow-up by CT were regarded as having inflammation. Patients who could not be diagnosed using bronchoscopy were diagnosed using surgical biopsy, repeat bronchoscopy, or CT-guided biopsy. Diagnostic yield was defined as the percentage of diagnostic cases.

### Assessment and statistics

To evaluate the safety and diagnostic utility, the incidence of complications and diagnostic yield were respectively compared between conventional biopsies and cryobiopsy using the McNemar-Bowker test for categorical data. In terms of diagnostic utility, the total diagnostic yield, which was the combination of each biopsy technique, was also compared with that of conventional biopsy alone. The diagnostic yield of conventional biopsy and cryobiopsy for each R-EBUS image obtained during the procedure was also compared.

Furthermore, factors affecting the occurrence of clinically significant bleeding (moderate, severe, and life-threatening) related to the procedure were analyzed using Fisher’s exact test. The clinical variables analyzed were chosen from those previously reported as risk factors for bleeding and empirically predicted factors as follows: patient age, sex, body height (≤ 170 cm or > 170 cm) [16], body mass index (≤ 25 kg/m^2^ or > 25 kg/m^2^) [26], lesion diameter (≤ 20 mm or > 20 mm), lobar position (right upper lobe/left upper segment, right middle lobe/left lingula, or bilateral lower lobes), location area (outer or inner), lesion appearance on CT [solid nodule or ground-glass nodule (GGN)], presence of the bronchus sign (positive or negative), the R-EBUS findings obtained (within, adjacent to, or invisible), presence of comorbid conditions [chronic obstructive pulmonary disease (COPD), ILD, and bronchial asthma], perioperative heparin bridging therapy (performed or not), cryoprobe size (1.9 mm or 2.4 mm), number of cryobiopsies performed (1 or ≥ 2), freezing time (≤ 5 s or > 5 s) [27], and sample size obtained by cryobiopsy (≤ 15 mm^2^ or > 15 mm^2^). Lesion diameter was determined based on the longest diameter on axial TSCT images. Location area was designated as “outer” if the lesion was in the outer-third ellipse or “inner” if the lesion was within the inner or middle third ellipse [19]. A GGN was defined as a peripheral pulmonary lesion with an area of increased attenuation and retention of the underlying vessels and bronchi on TSCT images [28]. The bronchus sign was a finding of the involved bronchus leading to the target lesion on TSCT [29]. The sample size was assessed using Olympus OlyVIA (version 3.2, Olympus) to enclose the biopsy section after scanning the hematoxylin and eosin (HE)-stained slides using a virtual slide system (VS-120, Olympus). These cut-offs were determined based on either our clinical experience (sample size) or previous reports.

Selected variables with *P* < 0.20 in univariable analyses were used in the analysis of multivariable logistic regression. Descriptive statistics expressed are numbers, percentages, and medians (range). All statistical tests were two-sided, and *P* < 0.05 was considered significant. All statistical analyses were performed using EZR (Saitama Medical Center, Jichi Medical University, Saitama, Japan), which is a graphical user interface for R (The R Foundation for Statistical Computing, Vienna, Austria) [30].

## Results

During the study period, 141 patients underwent transbronchial cryobiopsy using the two-scope technique for PPLs. Of them, cryobiopsy could not be completed in two cases because the tip of the cryoprobe could not be passed to the target lesion. The remaining 139 patients were enrolled and analyzed; a summary of patients’ characteristics are shown in Table 1. The median (range) size was 25.8 (8.7–72.7) mm. Most of the PPLs were located in the right upper lobe/left upper segment (50.4%) and at the outer area (61.8%). Lesions appearing on CT as solid nodules and as GGNs were seen at rates of 74.1% and 25.9%, respectively. Moreover, 20.1% had respiratory comorbidities (11.5% COPD, 4.3% ILD, and 4.3% bronchial asthma), and 3.6% received preoperative bridging anticoagulation therapy.

**Table 1:** Baseline characteristics of patients

Variable	N = 139 (100%)
Age (years)	72 (20–88)
Male	88 (63.3)
Body height (cm)	159.8 (140.0–182.6)
Body mass index (kg/m^2^)	22.6 (14.4–34.4)
Diameter of the lesion (mm)	25.8 (8.7–72.7)
Related bronchial generation into the PPL, generations	5 (3–8)
Lobar position	
Right upper lobe/left upper segment	70 (50.4)
Right middle/left lingula	17 (12.2)
Right lower/left lower	52 (37.4)
Location area	
Outer area	86 (61.8)
Inner area	53 (38.1)
Lesion appearance on CT	
Solid nodule	103 (74.1)
GGN	36 (25.9)
Bronchus sign on CT	
Positive	104 (74.8)
Negative	35 (25.2)
Bronchoscope type for Biopsy	
Thin scope	91 (65.5)
Thick scope	48 (34.5)
Respiratory comorbidities	
Chronic obstructive pulmonary disease	16 (11.5)
Interstitial lung disease	6 (4.3)
Bronchial asthma	6 (4.3)
Use of bridging anticoagulation therapy	5 (3.6)
Coagulation profile	
PT-INR	0.97 (0.87–1.17)
PTT (s)	25.9 (20.0–37.2)
Thrombocytes (× 10^3^/μL)	239 (113–524)

Data are presented as median (range) or n (%) values

*PPL* peripheral pulmonary lesion, *GGN* ground-glass nodule, *PT-INR* prothrombin time international normalized ratio, *PTT* partial thromboplastin time

**Fig. 2:**
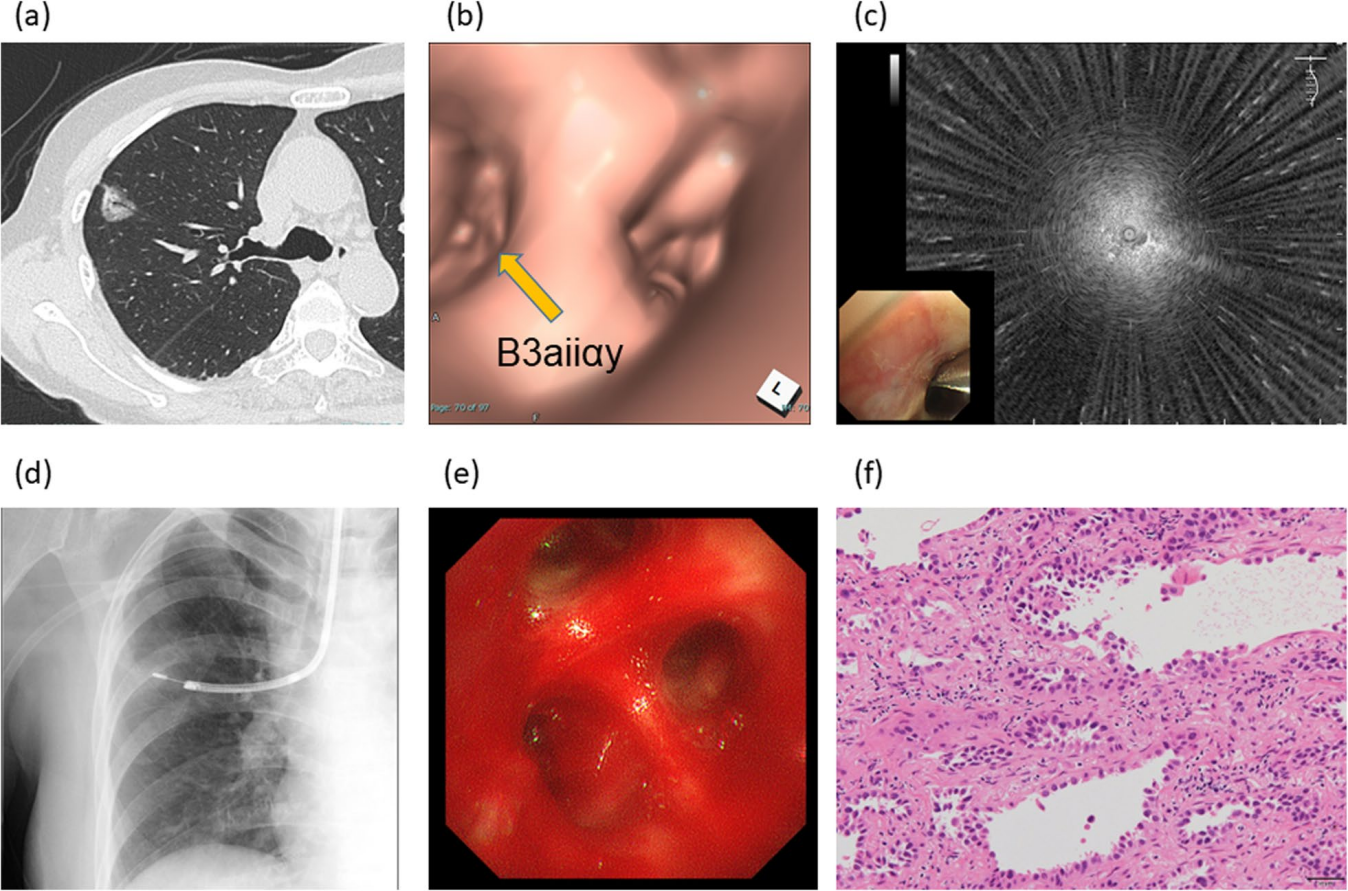
Representative case of transbronchial cryobiopsy using the two-scope technique for a peripheral pulmonary lesion. Computed tomography evaluation of an 82-year-old woman showing a part-solid ground-glass nodule (GGN) in the right upper lobe. CT shows it is located at right S3 (**a**). The target lesion is approached using a thin scope guided by virtual bronchoscopy displaying the bronchus route “B3aiiαy” toward the target lesion (**b**). Radial endobronchial ultrasound (R-EBUS) showing a blizzard sign (**c**). The biopsies using forceps and cryoprobe are performed with reference to fluoroscopy and R-EBUS imaging (**d**). Cryobiopsy causes moderate bleeding that is stopped using bronchoscopic suctioning in 2 min (**e**). Adenocarcinoma is revealed by only the cryobiopsy specimen (hematoxylin–eosin stain, × 200) (**f**)

The results of bronchoscopy are shown in Table 2. The median (range) procedure time was 28.2 (9.9–55.5) minutes, and the overall diagnostic yield was 89.9%. Figure 2 shows a representative case of successful transbronchial cryobiopsy using the technique for primary lung cancer.

**Table 2:** Results of bronchoscopy

	N = 139
Procedure time (min)	28.2 (9.9–55.5)
Bronchial generation of bronchoscope inserted, generations	4 (2–7)
EBUS images	
Within	52 (37.4)
Adjacent to	83 (59.7)
Invisible	4 (2.9)
Total diagnostic yield	125 (89.9%)

Data are presented as median (range) or n (%) values

*EBUS* endobronchial ultrasound

Regarding complications, conventional biopsies were not associated with any complications (Table 3). Although mild and moderate bleeding occurred in 42.4% and 18.0% of the patients, respectively, there were no cases of severe or life-threatening bleeding. Of the cases with moderate bleeding, five patients (3.6%) required thrombin instillation. The hemostatic bronchoscope entirely controlled all moderate bleeding episodes without requiring any other intervention. Cryobiopsy caused pneumothorax in two patients (1.4%), both of whom required thoracic drainage, an asthma attack in one patient (0.7%), and hypoxemia requiring oxygen inhalation for several minutes in three patients (2.2%). Of the three patients with hypoxemia, two were overlapped and caused by moderate bleeding, and the other was due to an asthma attack. There were no cases of pneumonia, lung abscesses, respiratory failure, or other severe complications.

**Table 3:** Complications during cryobiopsy using a two-scope technique for PPLs (N = 139)

Complication	Conventional biopsy	Cryobiopsy
Pneumothorax	0	2 (1.4%)
Bleeding		
Mild	0	59 (42.4%)
Moderate	0	25 (18.0%)
Severe/life-threatening	0	0 (0%)
Asthma attack	0	1 (0.7%)
Hypoxemia	0	3 (2.2%)

Data are presented as n (%)

*PPLs* peripheral pulmonary lesions

The clinical factors associated with clinically significant bleeding (moderate, severe, and life-threatening) with cryobiopsy are shown in Table 4. In univariable analyses, the ground-glass feature of the lesion was a significant factor for clinically significant bleeding (47.2% vs. 7.8%, *P* < 0.001). Sample size over 15 mm^2^ also tended to have a higher bleeding rate, but there was no significant difference (27.8% vs. 14.6%, *P* = 0.084). In multivariable analysis, only the ground-glass feature of the lesion had a significant effect (OR 9.30, 95% CI 3.40–25.40, *P* < 0.001) on the clinically significant bleeding rate compared to a solid feature. There were no significant differences between GGNs and solid nodules in the following factors: visualization yields on R-EBUS, within, adjacent to, and invisible in 30.6% (11/36) vs. 39.8% (41/103), 66.7% (24/36) vs. 57.3% (59/103), and 2.8% (1/36) vs. 2.9% (3/103), respectively, *P* = 0.66; median (range) number of cryobiopsy samples taken, 1 (1–2) vs. 1 (1–3), *P* = 0.26; and median (range) specimen size of cryobiopsy, 11.2 (3.3–29.1) vs. 11.4 (5.2–23.5), *P* = 0.87.

**Table 4:** Logistic regression analysis of clinical factors for clinically significant bleeding occurring with transbronchial cryobiopsy

Variable	Univariable analysis		Multivariable analysis	
	Incidence rate (%)	*P* value	OR (95% CI)	*P* value
Age (years)				
≤ 70	8/57 (14.0)	0.37		
> 70	17/82 (20.7)			
Sex				
Male	13/88 (14.8)	0.25		
Female	12/51 (23.5)			
Body height (cm)				
≤ 170	24/121 (19.8)	0.20	2.58 (0.29–23.30)	0.40
> 170	1/18 (5.6)			
Body mass index (kg/m^2^)				
≤ 25	22/103 (21.4)	0.13	2.63 (0.62–11.10)	0.19
> 25	3/36 (8.3)			
Diameter of the lesion (mm)				
≤ 20	10/47 (21.3)	0.49		
> 20	15/92 (16.3)			
Lobar position				
Right upper lobe/left upper segment	13/70 (18.6)	1		
Right middle/left lingula	3/17 (17.6)			
Lower	9/52 (17.3)			
Location area				
Inner area	9/53 (17.0)	1		
Outer area	16/86 (18.6)			
Lesion appearance on CT				
GGN	17/36 (47.2)	< 0.001	9.30 (3.40–25.40)	< 0.001
Solid nodule	8/103 (7.8)			
Bronchus sign				
Positive	18/104 (17.3)	0.8		
Negative	7/35 (20.0)			
R-EBUS image				
Within	7/52 (13.5)	0.41		
Adjacent to	17/83 (20.5)			
Invisible	1/4 (25.0)			
Chronic obstructive pulmonary disease				
Present	2/16 (12.5)	0.74		
Absent	23/123 (18.7)			
Interstitial lung disease				
Present	1/6 (16.7)	1		
Absent	24/133 (18.0)			
Bronchial asthma				
Present	1/6 (16.7)	1		
Absent	24/133 (18.0)			
Using bridging anticoagulation therapy				
Present	1/5 (20.0)	1		
Absent	24/134 (17.9)			
Cryoprobe size (mm)				
1.9	24/129 (18.6)	0.69		
2.4	1/10 (10.0)			
Number of cryobiopsies taken				
1 biopsy	24/120 (20.0)	0.20	0.29 (0.032–2.69)	0.28
≥ 2 biopsies	1/19 (5.3)			
Freezing time of cryobiopsy (s)				
≤ 5	12/59 (20.3)	0.66		
> 5	13/80 (16.3)			
Sample size (mm^2^)				
≤ 15	15/103 (14.6)	0.084	0.5 (0.17–1.46)	0.21
> 15	10/36 (27.8)			

*OR* odds ratio, *CT* computed tomography, *GGN* ground-glass nodule, *R-EBUS* radial endobronchial ultrasound

Table 5 presents the final histological diagnoses and diagnostic yields of each biopsy technique. Most of the PPLs were finally diagnosed as adenocarcinoma (61.2%). The diagnostic yields of conventional biopsy and cryobiopsy were 76.3% and 81.3%, respectively (*P* = 0.28). Of them, 8.6% (12 cases) were diagnosable only by conventional biopsy, and 13.7% (19 cases) were diagnosable only by cryobiopsy. When each biopsy technique was combined, the total diagnostic yield was 89.9%, which was significantly higher than conventional biopsy alone (*P* < 0.001). Although there was no significant difference in the diagnostic yield between conventional biopsy and cryobiopsy when R-EBUS images were “within” (conventional biopsy 49/52, 94.2% vs. cryobiopsy 44/52, 84.6%; *P* = 0.18) and “invisible” (conventional biopsy 1/4, 25.0% vs. cryobiopsy 1/4, 25.0%; *P*-value not available), the diagnostic yield of cryobiopsy was significantly higher than that of conventional biopsy when R-EBUS images was “adjacent to” (conventional biopsy 56/83, 67.4% vs. cryobiopsy 68/83, 81.9%; *P* = 0.019). In the 14 nondiagnostic cases, the diagnosis was established by surgery in six patients, repeat bronchoscopy in three patients, and CT-guided needle biopsy in one patient. The remaining four were clinically suspected to have lung cancer but were being followed-up on CT without surgery at the patients’ request.

**Table 5:** Histological diagnoses and diagnostic yield

	n	Conventional biopsy	Cryobiopsy	Total yield	*P* value
Malignant					
Adenocarcinoma	85	69/85	71/85	80/85	
Minimally invasive adenocarcinoma	1	0/1	1/1	1/1	
Adenocarcinoma in situ	2	0/2	1/2	1/2	
Adenosquamous carcinoma lung	1	1/1	1/1	1/1	
Non-small cell carcinoma	1	0/1	1/1	1/1	
Squamous cell carcinoma	21	17/21	17/21	19/21	
Small cell lung carcinoma	6	6/6	6/6	6/6	
Pleomorphic carcinoma	3	2/3	2/3	3/3	
Large cell neuroendocrine carcinoma	5	4/5	4/5	4/5	
Metastatic tumor	2	1/2	1/2	1/2	
Benign					
Screlosing hemangioma	1	0/1	1/1	1/1	
Organizing pneumonia	1	1/1	1/1	1/1	
Lung abscess	1	1/1	1/1	1/1	
Nontuberculous mycobacteria	2	2/2	2/2	2/2	
Chronic inflammation	2	1/2	2/2	2/2	
Cryptococcus	1	1/1	1/1	1/1	
Unknown^a^	4	0/4	0/4	0/4	
Total	139	106/139 (76.3%)	113/139 (81.3%)	125/139 (89.9%)	0.28

^a^These 4 cases are being followed-up by CT

## Discussion

The present study showed that the two-scope technique combined with cryobiopsy provides high safety, good hemostatic capacity, and high diagnostic yield (89.9%), which is associated with minimal complications from cryobiopsy for PPLs. Of 139 patients who underwent cryobiopsy using the two-scope technique, all bleeding episodes were completely controlled without additional bleeding control interventions, although moderate bleeding occurred in 25 patients (18.0%). Moreover, the lesion appearance on CT was shown to be a predictor of clinically significant bleeding after cryobiopsy, which may be helpful when selecting a biopsy technique.

Bleeding is a worrisome complication of cryobiopsy for PPLs, similar to ILDs [15–17]. In the present study, all bleeding episodes during cryobiopsy were controlled using the two-scope technique. This technique has the following advantages over the balloon method in terms of bleeding control. First, it allows easier hemostasis even in the bilateral upper lobe bronchi and B^6^, where balloon insertion is difficult [17]. Second, since there is no risk of balloon migration, further efficient endoscopic hemostasis can be achieved by combining it with postural change. Third, the amount of bleeding can be checked endoscopically in real-time, and hemostatic agents can be promptly added. Five cases with moderate bleeding required hemostatic agents and were easily controlled by prompt thrombin instillation in the present study. On the other hand, unlike ILD, cryobiopsy can be avoided in places with large blood vessels such as bronchial arteries by referring to R-EBUS images routinely checked in PPLs [31]. Furthermore, several (3–6) cryobiopsies from multiple areas and lobes are required to reduce sampling error in ILDs, whereas 1–2 specimens from one area are clinically sufficient for diagnosis of lung cancer and next-generation sequencing [32, 33]. In addition to effective hemostasis with the technique, the above differences in clinical backgrounds may have led to differences in the degree of bleeding between PPLs and ILDs with cryobiopsy. Accordingly, the results suggest that bleeding related to cryobiopsy for PPLs can be controlled using the two-scope technique. However, the preparation and immediate implementation of bleeding control interventions such as bronchial artery embolization and surgery are necessary for urgent life-threatening bleeding due to cryobiopsy [34].

One interesting finding was that the lesion appearance on CT was significantly associated with clinically significant bleeding. The present study did not find any significant relationship between clinically significant bleeding and short height and sex, previously reported as risk factors for bleeding with cryobiopsy of ILDs [16]***.*** The present findings are difficult to explain by referring to previous reports on bronchoscopy. However, this is in line with several previous reports concerning CT-guided biopsy that identified the ground-glass feature of the lesion as an independent predictive factor for more hemorrhagic complications [35–38]. These reports suggested that this is due to the preserved bronchovascular structures and less compact histological nature of GGNs, which show minimal histological changes, such as adenocarcinoma in situ, minimally invasive adenocarcinoma, and lepidic-predominant adenocarcinoma; preserved bronchovascular structures with less tumor invasion can increase the risk of iatrogenic access between an injured bronchus and pulmonary vessels associated with biopsy. Furthermore, the lack of compactness can reduce the compression effect on the biopsy site by the lesion itself [35]. These hypotheses can similarly explain our findings that the ground-glass feature of the lesion was a predictor of higher bleeding risk associated with cryobiopsy, which may help physicians decide whether to perform cryobiopsy.

Regarding other complications, pneumothorax occurred in 1.4% (2/141), whereas previous studies have reported a high incidence of pneumothorax in ILDs [39]. These two cases had PPLs located near the visceral pleura. The two-scope technique is free from the balloon blocker’s obstacle, allowing physicians to insert instruments into the correct bronchial route and obtain samples from the precise location for PPLs referring to VB and R-EBUS. Accordingly, the incidence rate of pneumothorax was lower than that previously reported for ILDs, and the complication rate, except for bleeding related to cryobiopsy, was maintained at the same level as in conventional biopsies. We presume that the complication rate of cryobiopsy for PPLs is comparable to that of conventional forceps biopsy, except for bleeding.

In the current study, most of the PPLs were detected using R-EBUS (97.1%, 135/139); however, the diagnostic yield of conventional biopsy remained at 76.3%. Thus, the diagnostic value of conventional biopsy has been insufficient due to its disadvantages (e.g., small crushed tissue specimen with artifacts, forceps’ weak mechanical strength of grasping, and collection site being limited in a forward direction). In contrast, cryobiopsy can harvest large specimens of good quality in a 360° manner laterally by freezing the surrounding peripheral lung tissue. Accordingly, 19 cases (13.7%) were diagnosed only by cryobiopsy, and the diagnostic yield when R-EBUS image was “adjacent to” and the overall diagnostic yield (89.9%) improved compared to conventional biopsy alone. Thus, cryobiopsy is considered a useful biopsy technique for diagnosing PPLs. However, 12 cases (8.6%) were diagnosed only be conventional biopsy. Presumably, the hardness of the cryoprobe tip caused misalignment in selecting the peripheral bronchus near the target lesion in these cases. The hardness caused interruption of cryobiopsy in two cases. Therefore, we assume that conventional biopsy and cryobiopsy play complementary roles in diagnosing PPLs. It may be better to perform both biopsy techniques to improve bronchoscopy diagnostic yield in PPLs at this stage. A thinner flexible cryoprobe, which has been recently developed and has shown good diagnostic utility in animal studies, may overcome the problems caused by the cryoprobe’s hardness and improve the diagnostic yield of cryobiopsy for PPLs in the future [40].

This study had several limitations. First, it was retrospective, non-randomized, and conducted at a single institution, increasing the probability of patient selection bias. Furthermore, multiple analyses may have occurred in multivariable analysis. It was difficult to estimate the risk factors for bleeding caused by cryobiopsy from previous bronchoscopy reports using conventional biopsy, which resulted in the increased number of clinical factors included in the analysis. Second, a direct comparison between the two-scope technique and the balloon occlusion method for cryobiopsy was not performed in this study. Finally, cryobiopsy was performed after conventional biopsy in all cases. The effect of conventional biopsy on bleeding related to cryobiopsy was not accurately evaluated. Further prospective studies are required to determine the optimal bleeding control technique for cryobiopsy for PPLs.

## Conclusions

The two-scope technique is a feasible hemostatic technique for transbronchial cryobiopsy for PPLs, providing safety and high diagnostic performance. We suggest that physicians should consider evaluating the lesion appearance on CT as a predictive factor for clinically significant bleeding related to cryobiopsy for PPLs.

## Data Availability

All data generated or analyzed during this study are included in this article. Further enquiries can be directed to the corresponding author.

## Abbreviations

CT: Computed tomography
PPLs: Peripheral pulmonary lesions
NSCLC: Non-small cell lung cancer
ILD: Interstitial lung disease
R-EBUS: Radial endobronchial ultrasound
GS: Guide sheath
VB: Virtual bronchoscopy
TSCT: Thin-section computed tomography
GGN: Ground-glass nodule
COPD: Chronic obstructive pulmonary disease
